# Supplementary material for: Analysis of PPARγ Signaling Activity in Psoriasis
Source: Int J Mol Sci. 2021 Aug 10;22(16):8603. doi: 10.3390/ijms22168603 (PMC8395241; doi:10.3390/ijms22168603)
Supplement: Supplementary file 1 [file ijms-22-08603-s001.zip › Supplemental materials_Analysis of PPARg signaling activity in psoriasis/Pathway models/Models images and html files/PPARG inhibits phenotypes assotiated with psoriasis/30365.html]

atherosclerosis


# Disease atherosclerosis

|  |  |
| --- | --- |
| URN | urn:agi-meshdis:Atherosclerosis |
| Total Entities | 13 |
| Connectivity | 9982 |
| Name | atherosclerosis |

---

|  |  |
| --- | --- |
| ChildConcepts | coronary atherosclerosis |
|  | carotid atherosclerosis |
|  | Binswanger's disease |
|  | cerebral atherosclerosis |
|  | intracranial atherosclerosis |
|  | atherogenesis |
|  | aortic atherosclerosis |
|  | atheromatosis |
|  | atherosclerotic plaque |
|  | atheroma |
|  | premature atherosclerosis |
|  | experimental atherosclerosis |
|  | atherosis |

---

|  |  |
| --- | --- |
| Pathway | Proteins with Altered Expression in Aging |
|  | Protein Oxidation and Nitration Products as Disease Biomarkers |
|  | Proteins Involved in Atherosclerosis |
|  | Insulin Resistance in Hepatocytes |
|  | Fatty Acids in Hypertension and Atherosclerosis Development |
|  | Tangier Disease Overwiew |
|  | CDKN2B-AS1 in Epigenetic Regulation of Glaucoma |
|  | PPARG inhibits phenotypes assotiated with psoriasis |
|  | efects selected |
|  | Neighbors of atherosclerosis |
|  | Figure 1\_Levothyroxine clinical effects |
|  | Figure 1\_Levothyroxine clinical effects |
|  | Overview. Diseases of circulatory system (chapter 8) |

---

|  |  |
| --- | --- |
| MedScan ID | 9010098 |

---

|  |  |
| --- | --- |
| Alias | Atheroslerosis |
|  | atheriosclerotic disease |
|  | Atherosclerosis |
|  | atheriosclerotic diseases |
|  | atheriosclerotic |
|  | Atheroscleroses |
|  | atheriosclerosis |
|  | Atherosclerotic Vascular Disease |
|  | atherosclerotic |
|  | Atherosclerotic Vascular Diseases |
|  | athero-sclerotic |
|  | athero-sclerosis |

---

|  |  |
| --- | --- |
| MeSH ID | D050197 |
|  | M0001903 |

---

|  |  |
| --- | --- |
| MeSH Heading | Atherosclerosis |

---
